# Background phase correction in congenital heart disease: does reliability vary based on underlying disease type?

**DOI:** 10.1186/1532-429X-18-S1-O94

**Published:** 2016-01-27

**Authors:** Sassan Hashemi, Senthil Ramamurthy, W James Parks, Denver Sallee, Tim Slesnick

**Affiliations:** 1 Cardiovascular Imaging Research Core, Children's Healthcare of Atlanta, Atlanta, GA USA; 2 Pediatrics, Emory University School of Medicine, Atlanta, GA USA

## Background

Phase-contrast magnetic resonance (PC-MR) allows non-invasive calculation of vascular flow, peak velocities and shunts. The technique, however, has inherent limitations, one of which is background phase errors. Various background phase correction (BPC) algorithms have been developed. The aim of this study is to apply various commercially available BPC algorithms in pediatric patients with a variety of disease types.

## Methods

Retrospectively, we analyzed patients in 4 categories: normal anatomy, stenotic aortic valve, dilated aortic root, and regurgitant pulmonary valve. All patients had PC-MR data obtained on a 1.5T magnet (Siemens MAGNETOM Aera) using a product sequence optimized for pediatric patients with free breathing techniques, multiple signal averages, and the vessel of interest placed at isocenter. We excluded patients with intracardiac shunting, arrhythmias or prosthetic valves. We calculated the aortic to pulmonary flow ratio (Qp:Qs) on 5 different analysis platforms both with and without BPC. Three platforms utilize a full-field stationary tissue fit and two others use a region of interest (ROI) for BPC, which was placed in stationary tissue as close to the vessel of interest as possible. One expert reader performed all vessel segmentations, ensuring all segmentations on different platforms used similar technique. Qp:Qs between 0.9 - 1.1 was defined as clinically acceptable.

## Results

**Figure 1:**
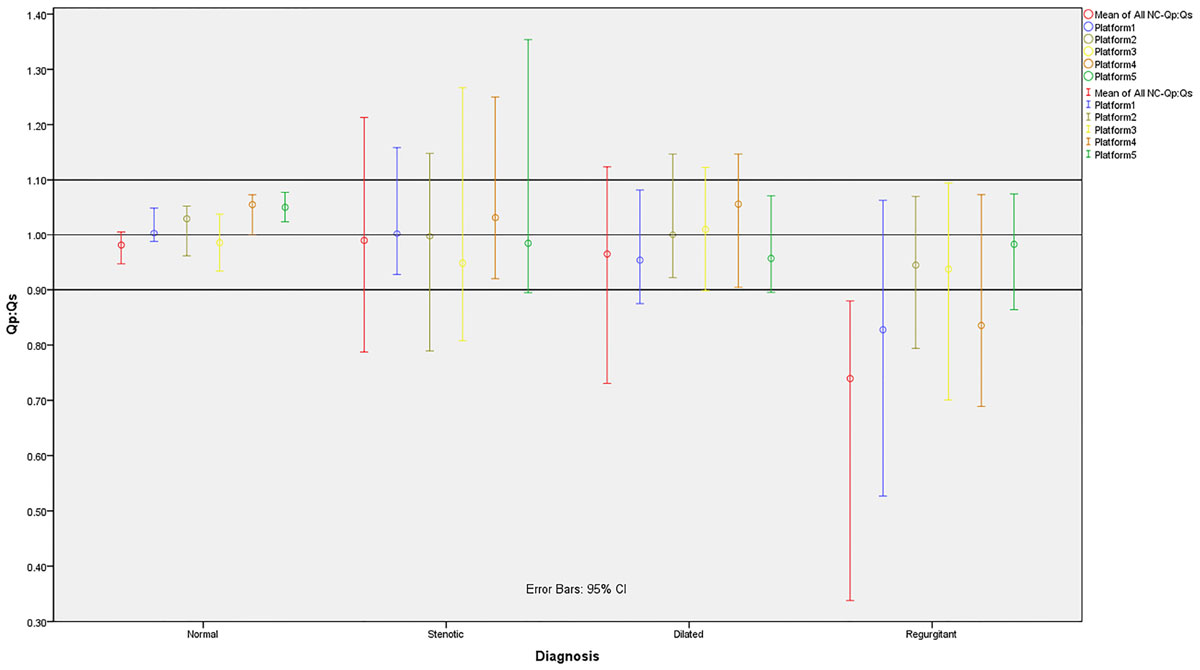
**Distribution of Qp:Qs in different categories for all platforms**. NC-Qp:Qs is the mean across all platforms. Data is represented as median and 95% confidence interval.

Fifty patients (76% males, mean age=13 ± 5 years) were analyzed (20 normal, 10 each from the other groups). The intraclass correlation coefficient for intra-observer reliability was 0.99. Distributions of Qp:Qs for different disease categories, before and after BPC, on all platforms are summarized in Table 1 and Figure 1. Non-corrected (NC) Qp:Qs in normal patients are distributed in the clinically acceptable range. The worst underestimation of Qp:Qs occurred in the regurgitant group, and BPC was significantly beneficial for these patients, with platform 5 being the most efficient. In the dilated aorta group, only the outlier Qp:Qs measurements benefitted from BPC, but the distribution of data were mostly in the acceptable range. BPC could not compensate for outlier measurements in the stenotic aortic valve group, though the medians of distribution did not deviate from 1.0.

## Conclusions

Non-corrected phase contrast values vary in clinical accuracy based on underlying disease type, and there are significant differences between various vendors' BPC algorithm efficiencies at resolving these discrepancies. These effects were most pronounced in pediatric patients with regurgitant lesions.

**Figure 2:**
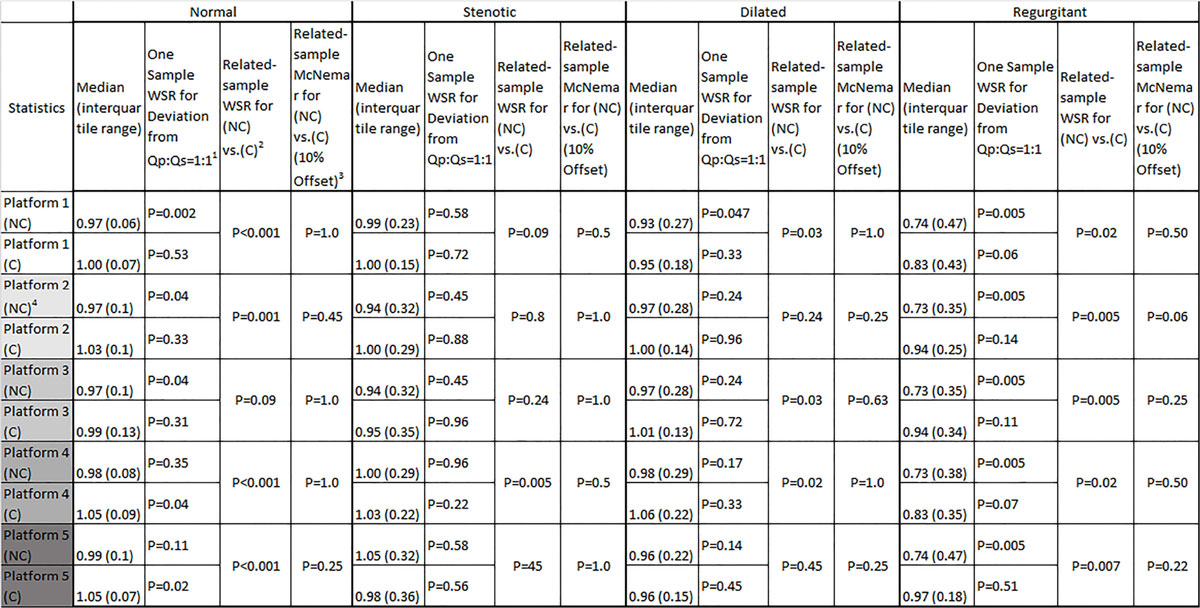
**(C): Corrected Qp:Qs, (NC): Non-corrected Qp:Qs, WSR: Wilcoxon Signed Rank Test**. 1. Null hypothesis: The median of Qp:Qs equals 1:1 (P < 0.05 rejects null hypothesis). 2. Null Hypothesis: The median of distribution between (NC) vs (C) equals "0" (P < 0.05 rejects null hypothesis). 3. Null Hypothesis: There is no significant difference between distribution of dichotomized Qp:Qs into clinically acceptable and not acceptable in (NC) vs. (C) (0.9:1.0 ≤ Qp:Qs ≤ 1.1:1.0 was set as clinically acceptable). 4. Platform 2 and 3 are two different background phase correction algorithms in a same post-processing package, thus sharing the same non-corrected data.

